# Improved Oxidative Stability and Sensory Quality of Beef Hamburgers Enriched with a Phenolic Extract from Olive Vegetation Water

**DOI:** 10.3390/antiox10121969

**Published:** 2021-12-09

**Authors:** Sara Barbieri, Dario Mercatante, Stefania Balzan, Sonia Esposto, Vladimiro Cardenia, Maurizio Servili, Enrico Novelli, Agnese Taticchi, Maria Teresa Rodriguez-Estrada

**Affiliations:** 1 Department of Pharmacy and Biotechnology, Alma Mater Studiorum-University of Bologna, 40127 Bologna, Italy; sara.barbieri@unibo.it; 2 Department of Agricultural and Food Sciences, Alma Mater Studiorum-University of Bologna, 40127 Bologna, Italy; dario.mercatante2@unibo.it (D.M.); maria.rodriguez@unibo.it (M.T.R.-E.); 3 Department of Comparative Biomedicine and Food Science, University of Padova, 35020 Legnaro, Italy; stefania.balzan@unipd.it (S.B.); enrico.novelli@unipd.it (E.N.); 4 Department of Agricultural, Food and Environmental Sciences, University of Perugia, 06126 Perugia, Italy; sonia.esposto@unipg.it (S.E.); maurizio.servili@unipg.it (M.S.); 5 Department of Agricultural, Forest and Food Sciences, University of Turin, 10095 Grugliasco, Italy; vladimiro.cardenia@unito.it; 6 Interdepartmental Centre for Industrial Agrofood Research, Alma Mater Studiorum-University of Bologna, 47521 Cesena, Italy

**Keywords:** beef hamburger, phenolic extract, olive vegetation water, olive by-product, clean label, lipid oxidation, cholesterol oxidation, sensory analysis

## Abstract

This study aims at evaluating the effect of a phenol-rich extract obtained from the concentration and purification of olive mill wastewaters (added at a ratio of 87.5 and 175 mg of phenols/kg meat) on the stability and sensory quality of beef hamburgers packed under modified atmosphere and stored under alternating exposure to fluorescent light at 4 ± 2 °C for 9 days. The hamburgers were sampled at different times (0, 6, and 9 days) and grilled at 200 °C. After 9 days, more than 56% of the added phenols in the raw burgers and more than 20% the grilled ones were retained. The results show that both concentrations of phenolic extract proved to effectively reduce primary and secondary lipid oxidation, as well as cholesterol oxidation products (COPs), during the shelf-life of raw hamburgers. Peroxide value, thiobarbituric acid reactive substances, and total COPs were up to 1.4-, 4.5-, and 8.8-fold lower in phenol-enriched raw hamburgers, respectively, than in the control samples; a similar trend was noted also in phenol-enriched cooked hamburgers (1.3-, 5.7-, and 4-fold lower). The sensory analysis also confirmed the effectiveness of the addition of phenolic extract, resulting in a positive effect on the red color intensity (raw product) and thus reducing browning during storage.

## 1. Introduction

The consumption of fresh ground meat preparations, like hamburgers, is wide-spread due to their pleasant taste and ease of cooking. The increasing demand for healthy and high-quality ground meat-based products has pushed the food industry towards the search for suitable strategies to render these products more stable during their shelf-life and thus limit changes of both color and flavor, two important factors that directly affect consumer’s acceptance [1]. In fact, one of the main problems that fresh ground meat preparations face during processing and storage is lipid oxidation, due to the cell membrane disruption during grinding and to their large surface/mass ratio. This is particularly true for ground red meat products, which have a high concentration of pigments (around 12%) that act as photosensitizers, as well as other oxidation catalyzing agents like cytochromes, non-heme iron, and other heavy transition metals [2]; moreover, the ferritin fraction can significantly promote lipid oxidation in heated meat systems [3]. Considering the high susceptibility of red meat to lipid oxidation, the International Agency for Research on Cancer (IARC) [4] has classified it in Group 2A (probably carcinogenic to humans), as it can give rise to a series of compounds (i.e., 4-hydroxy-nonenal (malondialdehyde)) that seem to contribute to adenocarcinoma formation and to the onset of colorectal cancer [5]. In addition, meat cholesterol can also undergo oxidation and generate cholesterol oxidation products (COPs), which have been associated with the development of several human diseases (i.e., cancer, cardiovascular, and neurodegenerative or autoimmune ones) [6,7,8]. To control lipid oxidation in fresh ground meat preparations and extend their shelf-life, food additives with antioxidant properties are usually employed. In a clean label perspective of meat product formulation, synthetic food antioxidants could be replaced by natural extracts obtained from agri-food by-products and waste, which are rich in bioactive compounds (such as carotenoids, phenolic compounds, essential oils, or β-glucans) [9,10] that display different health properties, and antioxidant and antimicrobial activities. In this regard, one of the most interesting food by-products is olive mill wastewater (OMWW), which is generated during olive processing for the production of olive oils and is characterized by a high content of organic compounds (sugars, tannins, pectins, and phenolic substances) and mineral salts [11]. This particular composition, together with its high generation rate, makes OMWW a highly polluting by-product whose uncontrolled disposal should be avoided; in particular, large quantities of polyphenols can exhibit marked antimicrobial, phytotoxic, and anti-nutritional properties and resistance to degradation, thus leading to negative effects on ecosystems [11]. However, OMWW could be considered an exploitable source of hydrophilic phenols (mainly secoiridoids, which are found exclusively in the *Oleaceae* family) with an antioxidant, antimicrobial, and anti-inflammatory activity [12,13]. Thus, phenols can be recovered from OMWW by using suitable membrane technology [14], for further applications in the food, pharmaceutical, or cosmetic industries [13,15,16]. Nevertheless, adding phenols from OMWW to extend ground meat products’ shelf-life requires careful dosage and sensory evaluation of newly formulated food products before market launch. In fact, phenolic substances affect bitter taste, pungent perception and astringency sensation [17,18], which may be negative drivers of liking [19]. 

Therefore, considering the need for prolonging the maintenance of quality during shelf-life in fresh ground meat preparations in a formulation perspective of clean label food products, this study aimed at evaluating the effect of the addition of an OMWW extract rich in phenols on the stability and sensory characteristics of raw and grilled beef hamburgers. Specifically, the main objectives were to verify the effectiveness of a powder formulation of the phenolic extract as a preservative agent for the extension of the shelf-life and the oxidative stability on the hamburgers, to define the sensory profile of these new phenol-enriched meat products and to monitor the perception of sensory characteristics and the presence of unacceptable sensory attributes and/or off-flavors during storage.

## 2. Materials and Methods

### 2.1. OMWW Phenol Extract

A crude phenolic extract (PE) was obtained by a three-step membrane filtration of fresh OMWWs, as previously reported [20]; the OMWWs derived from processing of olives from Moraiolo *cultivar*, which were harvested in Umbria (Central Italy). The PE was added with maltodextrin (1:1 d.w.) as a support, and then spray-dried to get a powder formulation of the PE.

### 2.2. Preparation of Phenol-Enriched Burger Samples

For the preparation of burgers, adult bovine meat (beef rump and shoulder muscle trimming) was obtained from animals reared and slaughtered in Spain, while the carcasses were sectioned in Italy according to Regulation EC/853/2004. The meat was trimmed and minced at a 6-mm diameter with a professional meat mincer (TCS32, Cavalli Meat Processing Machinery, Felino, Italy). The minced meat was mixed with salt (0.8 g/100 g) and starter cultures SafePro^®^ (B-SF-43, *Leuconostoc carnosum*) and Bactoferm^®^ (S-B-61, *Staphylococcus carnosus*) (Chr. Hansen GmbH, Germany), using a two-paddle mixer (IMP50-Bipala, Cavalli Meat Processing Machinery, Felino, Italy). The protective culture was added with the primary aim to control the spoilage bacteria (mainly *Enterobacteriaceae*) that can contaminate the beef meat; in this way, the unwanted variability factors that are not included in the experimental design (such as spoilage bacteria), are restrained. The dough was then divided into three batches: (i) control, meat dough plus maltodextrin (0.35 g/100 g, Maltodextrin Glucidex 19, Roquette, France); (ii) L1, meat dough plus PE equivalent to 87.5 mg phenols/kg of meat; and (iii) L2, meat dough plus PE equivalent to 175 mg phenols/kg of meat. Each batch was further mixed for 1 min and the burgers were then molded (about 80 g each patty), packed two per tray under modified atmosphere Alipak 333 mixture (50% nitrogen, 20% oxygen, and 30% carbon dioxide) and wrapped with a film made of polyethylene terephthalate (PET) + polyethylene (PE)/ethylene vinyl alcohol (EVOH)/PE (12 + 50 μm thickness, anti-fog and anti-UV; Guillin 5025N, Usmate-Velate, Italy). The trays containing the hamburgers were randomly divided and placed in a display refrigerator at 4 ± 2 °C for 9 days, under alternating exposure to fluorescent light (12 h light/12 h darkness) to simulate retail storage conditions. The burgers were sampled at fixed time periods (just after produced, T0; 6 days of storage, T6; 9 days of storage, T9) and frozen at −80 °C. At the same sampling times, the same number of hamburgers from each batch were grilled in an electrical grilling plate (Fimar FRY1L230M, Rimini, Italy) at 200 °C for 4 min per side until the core temperature reached 70 °C. For chemical analyses after cooking, burgers were cooled down at room temperature for 5 min, placed in a blast chiller (TBF051B, Moduline, Treviso, Italy) at −40 °C for 15 min, packed in a plastic bag under vacuum and stored at −80 °C until analysis. Two independent batches burger preparations were run.

### 2.3. Proximate Composition

Moisture, crude protein, and ash of raw samples were measured according to the AOAC Official Methods nos. 950.46.B, 981.10, and 920.15 [21]. Crude fat was calculated by difference, and the carbohydrate fraction was not taken into account. Measurements were carried out in duplicate.

### 2.4. Phenols Analysis

For the PE, 50 mg of spray-dried PE were solubilized in 10 mL of a methanol/water mixture (80:20, *v*/*v*), filtered with a 0.2 μm polyvinylidene fluoride (PVDF) syringe filter (Agilent Captiva, Agilent Technologies, Santa Clara, CA, USA) and injected into a high-performance liquid chromatograph coupled to a diode array detector (HPLC-DAD Agilent Technologies system Mod. 1100). The HPLC equipment and analytical conditions were those reported in [22]. Each measurement was done in duplicate.

For the hamburgers, 5 g of patties were mixed with 100 mL of methanol:water (80:20, *v*/*v*) containing 20 mg/L of butylated hydroxytoluene (BHT) + 0.2% trichloroacetic acid 1 M. The operations of homogenization, recovery, concentration until a final volume of 40 mL of extract, and purification of 10 mL of the aqueous extract by solid-phase extraction (SPE), were carried out as previously described [23]. The purified extract was then subjected to HPLC-DAD analysis using the same equipment and conditions as the PE analysis [22]. Each measurement was done in duplicate.

### 2.5. Chemical Analysis

#### 2.5.1. Lipid Extraction

The lipids were extracted according to Boselli et al. [24]. The extraction was performed on 5 g of hamburgers, which were added with 5α-cholestane (internal standard for the quantification of main lipid classes, see Section 2.5.2) (Sigma Chemical, St. Louis, MO, USA). The fat content was determined gravimetrically and expressed as percentage. Three independent replicates were run per sample.

#### 2.5.2. Determination of Main Lipid Classes

The qualitative−quantitative profile of the main lipid classes (free fatty acids, FFA; monoacylglycerols, MAG; free sterols, STE; diacylglycerols, DAG; esterified sterols, E-STE; triacylglycerols, TAG) was determined by gas chromatography-flame ionization detection (GC-FID), as reported by Gallina Toschi et al. [25] and Luise et al. [26]. An aliquot of 20 mg of the lipid extract dissolved in 1 mL of *n*-hexane was used for this analysis. The internal standard method, with the response factor of each main lipid class (estimated with commercial standards), was used to determine the amount of each lipid class (expressed as g/100 g of lipids). Three independent replicates were run per sample.

#### 2.5.3. Determination of Total FA 

The composition of total FA was determined on 20 mg of lipid extract by GC-FID [27], after previous methylation and transmethylation. FAME quantification was performed according to the internal standard method (using tridecanoic acid methyl ester) and was expressed as a proportion of the identified total FAME (g/100 g). Three independent replicates were run per sample.

Based on the total FA composition, the atherogenic index (AI) and thrombogenic index (TI) were also determined [28].

#### 2.5.4. Determination of Peroxide Value (PV)

PVs were determined in the lipid fraction extracted from hamburgers, using 10 g of meat and 50 mL of Folch solution [29]. After extraction, 10 mL of the recovered mixture was evaporated under nitrogen and used for PV determination by titration, according to Kim et al. [30]. The results were expressed as meq of active O_2_/kg meat.

#### 2.5.5. Determination of Thiobarbituric Acid Reactive Substances (TBARs)

Secondary lipid oxidation was assessed as TBARs on raw and grilled meat burger samples [31]. First, 2 g of each sample were used for this spectrophotometric determination and the absorbance was measured at 530 nm. A 1,1,3,3-tetramethoxypropane standard calibration curve was used for the quantification of TBARs (concentration range of 0.045–0.113 μg/mL; *y* = 0.0077 *x* + 0.0072, *r*^2^ = 0.9998) and the values were expressed as mg MDA/kg meat. Three independent replicates were made per sample.

#### 2.5.6. Determination of Cholesterol and its Oxides (COPs)

Cholesterol and COPs were extracted by cold saponification of 200 mg of lipid extract, followed by purification with aminopropyl SPE [27]. Silylated cholesterol and COPs were analyzed by Fast GC/MS [32], using betulinol (Sigma Chemical, St. Louis, MO, USA) and 19-hydroxycholesterol (Steraloids, Newport, RI, USA) as internal standards, respectively. Mass spectra were acquired in full scan mode (total ion current (TIC)), while they were integrated with single ion monitoring (SIM) mode using the characteristic ions with a high abundance [32]; quantification was carried out by means of calibration curves built for each compound. The cholesterol and total COPs were expressed as mg/kg of meat. Three independent replicates were run per sample. The rate of total cholesterol oxidation (%OR) was also estimated as reported by Cardenia et al. [27].

### 2.6. Physical Analysis 

#### Image Analysis

The instrumental measurement of appearance was carried out by an “electronic eye” (visual analyzer VA400 IRIS, Alpha MOS, France), a high-resolution CCD (charge-coupled device) camera (resolution 2592 × 1944 p) combined with powerful data processing software; the analysis was performed as described by Barbieri et al. [33]. The software application allowed to discriminate samples according to their different shape, size, color intensity, and color uniformity and, after building a data library, to process the data using multivariate statistical techniques. The image analysis using the electronic eye was performed on hamburgers before cooking in common purchase conditions.

### 2.7. Sensory Analysis

#### 2.7.1. Descriptive Analysis

The sensory quality of all samples (C, L1, and L2) at the three different storage times (T0, T6, and T9) was evaluated by a panel of 20 fully trained judges of both genders, aged between 20 and 65 years, and recruited on the basis of their previous experience in the field of sensory analysis and/or knowledge of product (staff and PhD students at the Department of Agricultural and Food Sciences, *Alma Mater Studiorum*-University of Bologna, Italy). A conventional profiling method was applied [34,35].

Sensory attributes were evaluated on a linear scale of 100 mm anchored at their extremes (0: absence of sensation; 100: maximum of sensation intensity); the results for five replicates and average values were calculated.

Regarding the sample’s preparation method, for the olfactory−retrolfactory, gustatory, and texture attributes, the burgers were evaluated after cooking (200 °C for a total of 8 min, 4 min for each side); for the visual attributes, the sensory analysis was performed on whole and raw samples, in order to mimic the normal conditions of the product purchase as much as possible. Moreover, to avoid judges influences by appearance and/or color of samples, the evaluation of the visual attributes was carried out at the end of the sensory evaluation. Finally, to standardize the tasting conditions and reduce bias, panelists evaluated visual attributes by observing the same sample inside a plate, whereas evaluation of other attributes (smell, taste, and texture) was performed by providing minced samples placed in plastic cups.

The samples were coded with three-random numbers and were presented to the assessors in randomized blocks with a break between samples.

#### 2.7.2. Discriminant Test

In this study, the triangle test was applied to identify sensory differences between the control sample (C) and the treated samples (L1 and L2) right after being produced (T0) and to investigate the effect of different storage times, i.e., 0, 6, and 9 days (CT0-CT6-CT9; L1T0-L1T6-L1T9, L2T0-L2T6-L2T9).

Since three samples at three different storage times had to be compared, the test was conducted in several successive sessions (two different days) to perform all possible combinations. The sessions were held in the tasting room of the Food Science Campus in Cesena (Department of Agricultural and Food Sciences, *Alma Mater Studiorum*-University of Bologna), involving 30 (first day) and 28 (second day) untrained judges aged between 20 and 65 years; the tests were carried out according to the procedures described by ISO 4120:2007. 

For the sample preparation, the same indications followed during the descriptive analysis were applied, except for the visual attributes, which, in this test, were evaluated on the cooked product. In each session, six triads of samples were evaluated. Data processing was performed by comparing the number of correct answers with values reported in a double entry probability table, indicating the minimum number of correct answers corresponding to the number of judges involved in the test or the number of judgments (number of judges for the number of replicas) for the different levels of significance [36].

### 2.8. Statistical Analysis

The software XLSTAT 7.5.2 version (Addinsoft, France) was used to elaborate the chemical, sensory, and physical data. 

The chemical data are reported as mean values of independent replicates of each analytical determination. First, normal distribution of data was tested (*p <* 0.05) with Shapiro−Wilk method. Chemical data were analyzed using two-way or three-way analysis of variance (ANOVA), including formulation (Form), storage time (St) and grilling (Gr) as factors, as well as their interactions (Form*St; Form*Gr; St*Gr; Form*St*Gr). Tukey’s honest significance test was performed at a 95% confidence level (*p* ≤ 0.05), to separate means of statistically different parameters. A principal component analysis (PCA) with a Varimax rotation was also carried out. 

For sensory data, significant differences among samples were evaluated by one-way and two-way ANOVA (multiple comparison test, Fisher LDS with *p* < 0.05). Moreover, to investigate the relationships between instrumental data (physical analysis) and the color of samples (sensory analysis), PCA and multiple factor analysis (MFA) were applied.

## 3. Results and Discussion

### 3.1. Proximate Composition

The proximate composition of the hamburger (with a moisture content of 70%, a crude protein content of 22%, and fat content around 5%) was roughly comparable to that of fresh lean meat (Table 1). The concentration of ash close to 2% was due to the salt addition during the dough mixing. No differences were detected among the different experimental batches, as well as between the beginning (0 days) and the end of the storage time (9 days). Such behavior confirmed that the film barrier prevented evaporation losses, which would have led to an increase in dry matter after 9 days of storage.

### 3.2. Evolution of Phenolic Compounds

The PE used for the preparation of the burgers had a total phenol content of 25.7 mg/g of dried product, of which 61.5% was 3,4-DHPEA-EDA (oleacein), 20.6% 3,4-DHPEA (hydroxytyrosol), 13.2% verbascoside, and 4.7% *p*-HPEA (tyrosol). 

As shown in Table 2, part of the added phenols was lost during storage and cooking. In terms of the total phenols, there was a loss of 34% and 43% of phenolic compounds in L1 samples after 6 and 9 days of storage, respectively, whereas a minor loss was detected in L2 burgers (16.2 and 36.5%, respectively). In particular, the highest variation was observed for 3,4-DHPEA-EDA, which decreased by 84% and 93% in L1 sample after 6 and 9 days, respectively, while in L2 its decrease was limited to 31% and 70%, respectively. Conversely, a significant increase in 3,4-DHPEA was detected (24.5% and 5.6% in L1 and L2, respectively) after 6 days; however, after 9 days, this compound was partly lost in L1, while it displayed a further increase in L2 burgers, reaching a total positive variation of 19%. 

As the increase of 3,4-DHPEA in presence of 3,4-DHPEA-EDA has already been observed in different food matrices and at different temperatures [37,38,39,40], the phenols evolution in L1 and L2 hamburgers strengthened the hypothesis of its hydrolytic origin from the degradation of oleuropein derivatives during storage [41]. Nevertheless, the oxidative degradation of these two phenols has been appointed as the main cause of their decrease [42,43]. For hydroxytyrosol, therefore, two contemporary phenomena would be at the basis of its particular evolution over time: the first is the hydrolysis of 3,4-DHPEA-EDA after which this phenolic alcohol is released in free form, while the second is the oxidative degradation, which leads to its decrement. In the case of samples L1 and L2, in the early stages of storage, hydroxytyrosol was limitedly involved in oxidation reactions and, therefore, the resultant balance between the decrease on the one hand and the increase on the other was an increase of the concentration of hydroxytyrosol. In the more advanced phases, the oxidative degradation prevailed in the L1 hamburgers, due to the decrease in the concentration of the other more reactive phenols (such as 3,4-DHPEA-EDA); in the L2 samples, on the contrary, with the concentration of 3,4-DHPEA-EDA still being high until the ninth day of storage, the balance was evidently still in favor of the increase of 3,4-DHPEA by hydrolysis rather than its decrease by oxidation.

During storage, the concentration of *p*-HPEA and verbascoside did not significantly vary, as already found in other shelf-life studies for food matrices [44]. Despite the significant decrease during storage, in terms of the total polyphenols, the percentage retained was 56.5% and 63.5% of the initial amount added to L1 and L2, respectively.

Regarding the behavior of phenolic compounds during cooking, the loss was greater than those found during storage. In all grilled samples, 3,4-DHPEA-EDA completely disappeared, confirming its high susceptibility to high cooking temperatures [45], while a severe loss of hydroxytyrosol (ranging between 53 and 100% from 0 to 9 days) was observed as well. Differently from the evolution noticed during storage, *p*-HPEA and verbascoside also showed a consistent decrease with grilling. However, even after cooking, the amount of the retained phenols ranged between 12.4% (L1 T9) and 29.4% (L2 T0).

### 3.3. Chemical Analysis

#### 3.3.1. Lipid Content and Main Lipid Classes 

As reported in Table 3, the lipid content ranged from 4.9 to 6.3% and from 6.1 to 7.5% in raw and grilled burgers, respectively. The formulation and storage did not significantly affect the lipid content of both raw and grilled samples. However, it was possible to note a significant increase in the lipid amount of the grilled samples, which could be attributed to the free water loss during grilling, with consequent dehydration of the meat product [46]. 

Regarding the main lipid classes (Table 3), the most abundant class was TAG, followed by FFA, DAG, E-STE, STE, and MAG in both raw and grilled burgers. While the shelf-life did not significantly affect the percentage distribution of the single lipid classes, the formulation apparently had an effect on the FFA, MAG, STE, E-STE, and TAG, but without displaying a clear trend. These slight differences could be partly due to the non-homogeneous nature of hamburgers, as they were prepared with minced beef meat from different cuts. After cooking, some significant differences were detected in the total lipid profile (in particular MAG, DAG, and STE), which may have been influenced by a combined effect of lipolysis and a partial/selective loss of the lipid components during grilling, due to their different melting points.

#### 3.3.2. Total Fatty Acid Profile 

About the total FA composition (Table 4), the most represented FA class was monounsaturated FA (MUFA, 55–58%), followed by saturated FA (SFA, 36–42%) and polyunsaturated FA (PUFA, 2–4%). This FA profile agrees with that reported by Gruffat et al. [46] for beef meat.

No significant effect of preservation time on the FA profile of hamburgers was observed (Table 4). As expected, PE addition significantly stabilized PUFA in raw hamburgers (in particular PUFA *n-6*), which could be mainly ascribed to the antioxidant activity of the remaining hydroxytyrosol and oleacein (≈73% of the total remaining phenols; Table 2). However, grilling significantly affected the single PUFA classes, but no defined trend was observed. In addition, a significant interaction among the product formulation, storage time, and grilling was noted only for PUFA and PUFA *n-6*. In this study, grilling may have induced modifications in the FA composition of hamburgers through the oxidation of unsaturated FA and/or selective melting of lipids [47]; these two effects added up to the selective degradation of phenols in the grilled PE-enriched products (Table 2), as oleacein completely disappeared after cooking.

Regarding FA class ratios (Table 5), the PUFA *n*-6/PUFA *n*-3 ratio varied between 3.59 and 5.45 in raw hamburgers, while it ranged from 3.99 to 5.85 in grilled ones; these values agreed with those found by Gruffat et al. [46] for raw and grilled beef meat. According to Simopoulos [47], a low PUFA *n*-6/PUFA *n*-3 ratio (<4) is desirable for a healthy human diet, so product formulation should consider this parameter. Grilling significantly impacted this ratio as a consequence of its effect on the single PUFA classes, but no common trend was observed among the different types of samples. A significant interaction between the product formulation and storage time was detected for this ratio.

The UFA/SFA ratio is useful for observing the oxidative stability of FA in food, as it decreases when UFA oxidizes. The UFA/SFA ratio ranged from 1.40 to 1.73 in raw burgers, while it varied from 1.36 to 1.66 in grilled samples. These values agree with those reported by Gruffat et al. [46] for raw and grilled beef meat. None of the factors here tested (product formulation, storage time, and grilling) significantly influenced this ratio. 

The PUFA/SFA ratio is also used for evaluating the nutritional quality of food lipids, and it has been suggested by nutritional guidelines that it should be around 0.4. In our case, this ratio ranged between 0.05 and 0.12 in raw hamburgers, whereas it varied from 0.06 to 0.30 in the grilled ones. The results of the present study agree with the PUFA/SFA ratio reported by Gruffat et al. [46] in raw and after grilling the beef meat. This ratio reflected the behavior of the PUFA class, as it was also significantly influenced by formulation and showed a significant interaction among the product formulation, storage time, and grilling.

Based on the FA composition, AI and TI were also calculated, which are useful indices for understanding the role of FA composition on both atherogenic and thrombogenic risks. The indices ranged from 0.53 to 0.69 and from 1.08 to 1.41 for AI and TI, respectively, which agreed with those reported by Gruffat et al. [46] for beef meat cooked using diverse techniques. While the TI values were not significantly different, AI was significantly affected by the formulation, confirming the protective effect of PE on unsaturated FA mainly observed in raw hamburgers. A significant interaction among the three factors (product formulation, storage time, and grilling) was also detected in AI. 

Regarding single FA, the most abundant FA in both raw and grilled samples was oleic acid (C18:1 *n*-9), followed by palmitic acid (C16:0) and stearic acid (C18:0) (Table S1 Supplementary Materials). No significant differences were found in the single FA between control and PE-enriched samples during shelf-life. Grilling significantly decreased the content of palmitoleic acid and its *trans* isomer (C16:1 *n*-7 and C16:1*t* *n*-7)*,* as reported by Gruffat et al. (2021).

#### 3.3.3. Lipid Oxidation

Primary lipid oxidation products were monitored by PV, while TBARs and total COPs were determined as the secondary oxidation products (Table 6).

PV ranged from 0.84–5.63 and 5.65–17.74 meq O_2_/kg of fat in raw and grilled samples, respectively. Product formulation, storage time, and grilling significantly influenced this oxidative parameter; a significant interaction between storage and grilling was also noticed. All of this resulted in PV data that were up to 1.4- and 1.3-fold lower in phenol-enriched raw and cooked hamburgers, respectively, than in the control samples. Significantly lower PV levels (0.84–3.58 and 5.65–13.12 meq O_2_/kg of fat in raw and grilled samples, respectively) were found in L2 samples, with 175 mg phenols/kg added to the meat; in fact, a PE concentration-dependent effect was observed in grilled hamburgers that had previously been stored for 6 and 9 days. Preliminary lipid oxidation may have already been initiated during meat mincing, as grinding was carried out in the presence of air, and oxygen availability is known to be particularly critical for highly pigmented meats as beef; the salt addition during dough mixing might have also exerted a pro-oxidant effect [48]. Despite being packed under a modified atmosphere, the latter contained 20% oxygen, which might have contributed to further formation and accumulation of hydroperoxides during storage. However, due to their high instability, the hydroperoxides greatly decomposed when hamburgers were grilled, giving rise to free radicals species that led to propagation of lipid oxidation. In any case, due to their free radical scavenging action [23], the added phenols demonstrated to efficiently contrast the formation of lipid hydroperoxides during storage and after grilling, despite the selective degradation of phenols in the grilled PE-enriched products (Table 2).

When hydroperoxides decompose, they give rise to alkoxyl and hydroxyl radicals, which can evolve into secondary oxidation products such as aldehydes and ketones, whose presence can be determined as TBARs. In the present study, TBARs ranged from 0.48–4.86 and 0.52–4.07 mg MDA/kg in raw and grilled burgers, respectively, and were up to 4.5- and 5.7-fold lower in phenol-enriched raw and cooked hamburgers, respectively, than in the control samples. Product formulation and storage time significantly affected TBARs, and the interaction between these two factors was significant as well. A PE concentration-dependent effect was observed in both raw and grilled samples. As observed for PV, PE was also able to limit the formation of secondary oxidation products during storage and after grilling, in spite of cooking-induced pro-oxidant conditions that arise from iron release from denaturated heme pigments (chiefly myoglobin) [49]. In our study, the PE added to the hamburgers did not lose its effectiveness after grilling thanks to its formulation with the addition of maltodextrins during spray-drying, which exerted a protective role. In fact, TBAR values of phenol-enriched raw and grilled samples were below or very close to 1 mg MDA/kg, value above which rancid flavor begins to develop in meat [50]. Our TBARs results were similar to those reported by Aouidi et al. [51] and Shalaby et al. [52] in beef burgers formulated with a phenol rich extract derived from olive leaf in a ratio of 105 mg of PE/100 g of minced beef. In the study of Aouidi et al. [51], the antioxidant activity of olive leaves extract against lipid oxidation was lower in cooked meat than in raw one, which was attributed to the inhibition of the antioxidant activity of olive leaves extract by substances produced during cooking. 

On the other hand, cholesterol is an important constituent of cell membranes and, as UFA, it is also susceptible to oxidation. The total content of cholesterol (Table 6) ranged from 538.27 to 926.07 mg/kg in raw hamburgers and from 1140.97 to 1225.14 mg/kg in grilled ones. These data agree with those reported by Barriuso et al. [53] for beef hamburgers. Cholesterol content was not significantly affected by the addition of the phenolic extract, nor by the storage time. However, grilling significantly increased the cholesterol content of the hamburgers, which could be ascribed to the water loss and consequent dehydration of the product during grilling [46].

Regarding total COPs, they varied from 0.90 to 6.32 mg/kg in raw hamburgers and 2.9 to 17.0 mg/kg in cooked ones (Table 6). The profile and amount cholesterol oxides detected in the present study were similar to the one reported by Barriuso et al. [53] for raw and cooked beef burgers, where oxidation products deriving from the monomolecular reaction pathway (i.e., 7-derivatives) were more abundant than those generated by bimolecular ones (i.e., epoxy and triol derivatives); in particular, triol was present at very low levels (<0.3 mg/kg) in the grilled samples, whose formation is known to be favored by water in acid conditions. In the present study, product formulation, storage time, and grilling significantly influenced the total COPs, and all the two-factor and three-factor interactions were significant as well. Cholesterol oxides displayed a similar behavior to PV and TBARs, as the total COPs were up to 8.8- and 4-fold lower in the PE raw and PE cooked hamburgers, respectively, than in the control samples, thus confirming a PE concentration-dependent effect related to their chain-breaking antioxidant activity.

The cholesterol oxidation ratio (COR) ranged from 0.12% to 1.51% and from 0.12% to 1.08% in the raw and grilled samples (Table 6), respectively, which was significantly higher in control samples than in phenol-enriched ones. Similarly to the total COPs, the product formulation, storage time, and grilling significantly influenced this ratio, but only Form * St and the three-factor interaction were significant. While both control and L1 showed a constant increase of COR during storage, L2 exhibited a steady level of COR. In addition, the COR of the majority of cooked PE hamburgers was about two times higher than their corresponding raw samples. 

As it emerged from the study, the use of phenol extracts from agricultural by-products allowed for limiting lipid oxidation and the formation of potentially harmful compounds such as COPs [6,7,8]. Successful results were also obtained by using a phenolic extract rich in 3,4-DHPEA, *p*-HPEA, VB, and 3,4-DHPEA-EDA in raw and cooked pork sausages [40].

#### 3.3.4. Principal Component Analysis (PCA) of Chemical Data

**Figure 1:**
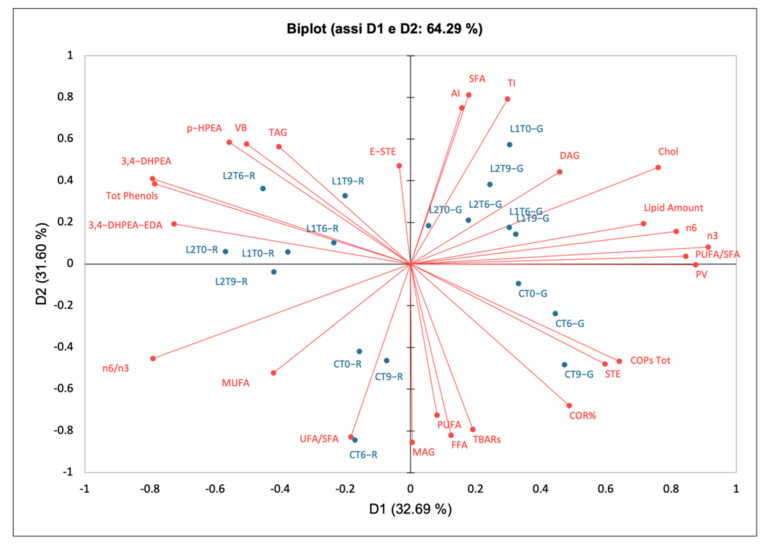
Biplot of raw and grilled hamburgers. 3,4-DHPEA, Hydroxytyrosol; 3,4-DHPEA-EDA, decarboxymethyl oleuropein aglycone; AI, Atherogenic Index; COPs, cholesterol oxidation products; COR, cholesterol oxidation ratio; DAG, diacylglycerols; E-STE, esterified sterols; FFA, free fatty acids; MAG, monoacylglycerols; MUFA, monounsaturated fatty acids; *p*-HPEA, tyrosol; PV, peroxide value; PUFA, polyunsaturated fatty acids; SFA, saturated fatty acids; STE, sterols; TAG, triacylglycerols; TBARs, thiobarbituric acid reactive substances; TI, thrombogenic index; UFA, unsaturated fatty acids; VB, verbascoside.

To better understand which parameters were the most relevant for assessing the effects of phenolic enrichment, cooking treatment, and storage on the hamburgers, the chemical and phenolic composition data were subjected to principal component analysis (PCA) for raw and grilled hamburgers (Figure 1). 

The first two components explained 64.29% of the total variance (32.69% for PC1 and 31.60% for PC2). All of the oxidative parameters (COPs, TBARs, COR, and PV) are in the opposite quadrant (3) with respect to the total phenols and the single phenolic compounds (3,4-DHPEA, 3,4-DHPEA-EDA, *p*-HPEA, and VB; quadrant 1).

The grilled samples were well separated from the raw ones and were located in different quadrants. Control grilled samples were more correlated to COPs and COR, while the L1 and L2 grilled samples were more related to DAG. This correlation could be explained by the fact that the phenolic extract present in L1 and L2 was able to limit cholesterol oxidation, but not TAG hydrolysis.

The control raw samples were more correlated with the UFA/SFA ratio, while L1 and L2 raw samples were more related to the total and single phenols. This could be due to the susceptibility of phenols to high temperatures, which led to a greater loss of phenols in L1 and L2 grilled samples compared to the corresponding raw samples over the shelf-life (Table 2). In all grilled samples, in fact, 3,4-DHPEA-EDA completely disappeared.

These results confirm that the addition of PE can limit the oxidative phenomena in both raw and grilled hamburgers. Although grilling tends to decrease the phenol content, these are still able to exert an antioxidant activity. In fact, as described in Section 3.2, even after cooking, the amount of phenols retained by hamburgers ranged between 12.4% and 29.4%.

### 3.4. Physical Analysis

#### Image Analysis

The electronic eye was applied to support the development of the sensory profile of the hamburgers; it performs the analysis of the product appearance through continuous image acquisition in extremely short time. The acquired images of burger samples (C, L1, and L2) stored at three times (0, 6, and 9 days) were processed using the Alphasoft software (version 14.0).

**Figure 2:**
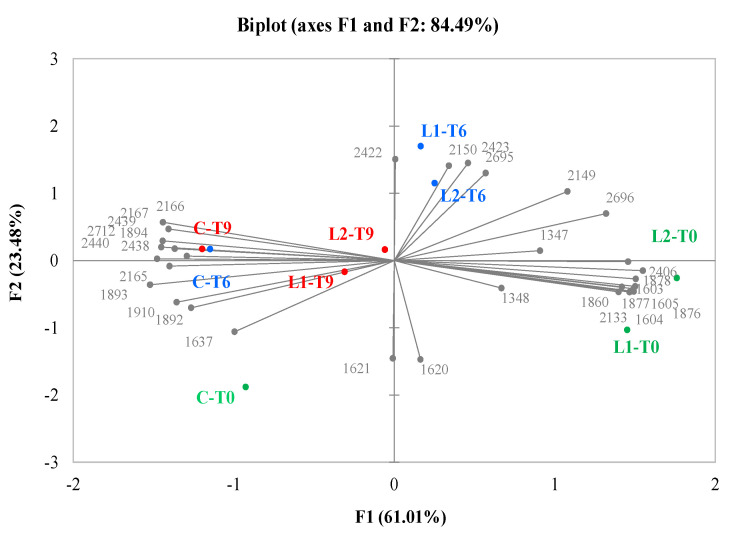
Representation of the cases and variables obtained from the PCA related to the results of the image analysis (electronic eye) for the three samples under examination (C, Control (minced beef meat + maltodextrine + starter cultures); L1, minced beef meat + starter cultures + 87.5 mg phenols/kg of meat; L2, minced beef meat + starter cultures + 175 phenols/kg of meat) evaluated at all of the storage times (T0, T6, and T9).

Samples and instrumental (electronic eye) data were subjected to PCA (Figure 2) and projected into a two-dimensional plane composed of four quadrants to highlight possible correlations. The first two components explained 84.49% of the total variance (61.01% for F1 and 23.48% for F2). The different direction of vectors (loading PCA) showed the variables (colors) involved in the variations of appearance among samples. By analyzing the different positions of the samples on the surface (PCA score), it was possible to observe that the control samples (CT0, CT6, and CT9) were more characterized by the variables present between the second and third quadrant that described the greatest color intensity and influenced the position of the samples with a brown/gray color. This would therefore indicate that sample C was more oxidized; the brown color, in fact, could be due to a prevalence of metmyoglobin, the oxidized form of myoglobin. On the contrary, the phenol-enriched samples at time 0 were close to the variables that described a low color intensity (fourth quadrant), that is a color that tends more to red. In this case, however, the addition of PE in the samples tended to create a reducing environment, with a consequent prevalence of oxymyoglobin, responsible for the bright red color of the sample. On the other hand, an intermediate color characterized the phenol-added samples stored at 6 and 9: they tended to be lighter for L1T6 and L2T6 (first quadrant) and darker for L1T9 and L2T9 (second and third quadrant). It can therefore be deduced that the presence of phenolic extracts and bioprotective cultures contributed to improving the visual quality of the burgers at both time 0 and during storage.

### 3.5. Sensory Analysis

#### 3.5.1. Descriptive Analysis

The sensory profile of the phenol-enriched burgers was obtained by applying the QDA^®^ method. The final list of descriptors included: (i) two relative to appearance (presence of fat/connective tissue (total amount of fat/connective tissue inside the whole hamburger), and color intensity (from red to brown/gray)); (ii) two perceived by orthonasal and retronasal routes (beef flavor (flavor associated with cooked beef), and bloody (flavor associated with blood or raw beef)); (iii) one gustatory (salty-basic taste); and (iv) two relative to the texture (tenderness (how easily it is chewed or cut) and juiciness (amount of juice released from the product during mastication)).

When the training was completed, the sensory evaluation was carried out by the panel in five replicates for each type of sample at three different storage times (frozen just produced, T0; frozen after 6 days of storage, T6; frozen after 9 days of storage, T9). The average values for each attribute assessed are reported in Table 7.

Concerning the control samples (C), we noticed that there were no statistically significant differences in the attributes assessed by the panel during product storage, except for the juiciness and color intensity. In particular, the juiciness decreased passing from 6 to 9 days of storage, while between T0 and T6, no differences were perceived for this texture attribute; the reduction of juiciness could be attributed to a decrease of water holding capacity related to protein denaturation/oxidation during storage. The intensity of the color (from red to brown/gray), instead, showed the opposite trend, increasing significantly after 6 days of storage and thus confirming that myoglobin oxidation is already detectable after this time. The PE addition in sample L1 (87.5 mg phenols/kg of meat) did not affect most of the sensory attributes during storage; the only changes were related to the juiciness and tenderness texture attributes: in both cases, a decrease was observed over time. Unlike the control sample, L1 did not show any differences in color intensity. Since L2 had a higher concentration of phenolic extract (175 mg/kg of phenols), it displayed statistically significant differences only for the color intensity attribute, which significantly increased after 9 days of storage.

Considering the comparison among samples at the diverse storage times, it is possible to note that for samples just produced (T0), the addition of the phenolic extract resulted in a color variation that showed lower values in both samples L1 and L2. In fact, the panel did not highlight any changes in the other attributes evaluated in the olfactory-retro–olfactory, gustatory, and texture phases.

After 6 and 9 days of storage, the effect of the addition of phenolic extract on the meat color was confirmed: for both storage times a decrease in color intensity (0: red; 100: brown/gray) was found in the two phenol-enriched samples compared with the control one. Furthermore, the intensity of the beef flavor was higher in L1 and L2 compared to C, confirming the positive effect on the product performance during storage.

**Figure 3:**
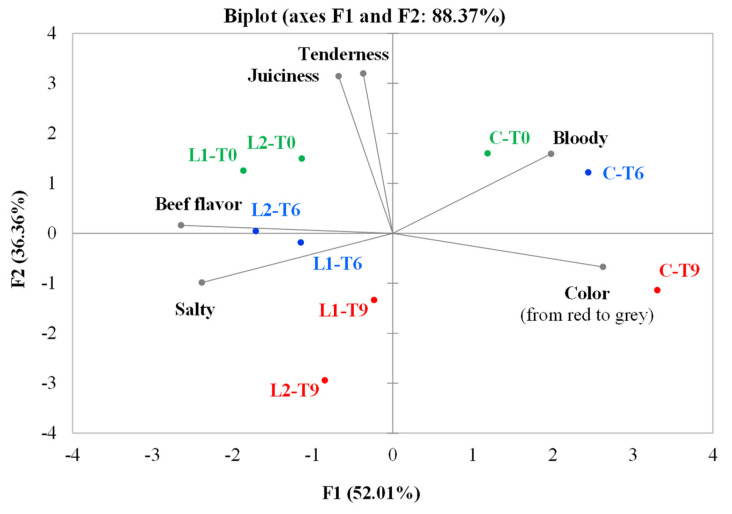
Representation of the cases and variables obtained from PCA related to the results of the QDA^®^ of the three samples under examination (C, control (minced beef meat + maltodextrine + starter cultures); L1, minced beef meat + starter cultures + 87.5 mg phenols/kg of meat; L2, minced beef meat + starter cultures + 175 phenols/kg of meat) evaluated at all the storage times (T0, T6, and T9).

Samples and sensory attributes (most significant according to ANOVA) evaluated by the panel were projected into a two-dimensional plane composed of four quadrants to highlight possible correlations by PCA (Figure 3). 

The first two components explained 88.37% of the total variance (52.01% for PC1 and 36.36% for PC2). The control samples at 0 and 6 days of storage (CT0 and CT6) were located in the first quadrant and characterized by a strong blood aroma and high tenderness. The fresh phenol-enriched samples (L1T0 and L2T0) were placed in the second quadrant, whose position was influenced by the high intensity of juiciness and beef perceived by the panel. The samples at 6 days of storage (L1T6 and L2T6) had similar characteristics were between the second and the third quadrant, being characterized by a low color intensity (0: red; 100: brown/gray). After 9 days of storage, the L1 and L2 samples positioned in the third quadrant: although these samples were characterized by a high intensity of color compared to those tested at time 0 and 6 days, they showed a reduction of juiciness, tenderness, and beef flavor, as well as an increase in saltiness. Finally, the control sample at time 9 days is located in the fourth quadrant, which was very similar to the C at 0 and 6 days, but less tender and juicy and with a dark color (high color intensity).

Besides the evaluation of the descriptors according to a linear scale, assessors were asked to indicate the presence of off-flavors and negative sensations. After elaborating these comments, the presence of olfactory−gustatory anomalies (fermented, oxidized, and rancid) emerged for the control sample at time 6 and 9 days, while these anomalies were not significant for both L1 and L2.

#### 3.5.2. Discriminant Test

The triangle test was carried out to verify the existence of significant differences between the two phenol-enriched hamburgers (L1 and L2) and the control sample (C), and among each type of sample stored at different times (0, 6, and 9 days), in order to provide useful information to evaluate the shelf-life of the new formulated products. The test was organized in several sessions in which all possible sample combinations were presented to the subjects (Table 8).

Sessions 1–3 aimed to compare all samples (C, L1, and L2) at time 0 and the results, obtained from the 30 subjects, showed significant differences. In particular, the L1T0 sample was recognized as different from CT0 because it was characterized by a more intense beef flavor and different texture (session 1), while the CT0 sample differed from L2T0 by the less intense meat flavor and juiciness (session 2). The L2T0 sample was discriminated from L1T0 for the lower intensity of beef flavor, tenderness and juiciness (session 3). These results agree with those of Balzan et al. [40], where the subjects involved were able to discriminate between the three formulations (C, L1, and L2); however, the color was never mentioned as a discriminating factor, even though the color variations were significant, especially for the raw product, as evidenced by the physical and sensory results previously discussed. On the other hand, the phenol-enriched hamburgers (L1 and L2) were perceived as being significantly different. 

In sessions 4–6, a comparison between the control samples at the three storage times, was carried out. Only the session in which CT0 was compared with CT6 and CT9 evidenced significant differences, while no differences between CT6 and CT9 were detected (session 6). When CT0 and CT6 were compared (session 4), participants indicated that differences were related to a lower intensity of beef flavor (sometimes considered unpleasant and/or anomalous), tenderness, and juiciness of the sample stored for 6 days. In the comparison between CT0 and CT9 (session 5), the judges correctly identified the different sample (CT9), because it was saltier, more acid, harder, and drier than CT0 and it was characterized by an unpleasant taste (sometimes indicated as rancid and/or “expired”).

Further sessions were conducted for the evaluation of the phenol-enriched samples at the three storage times. Interviewees (*n* = 28) found statistically significant differences between L1T0 and L1T9 (session 8) due to the lower overall taste (tasteless) of sample L1 at time 0, whereas the differences between L1T6 and L1T9 (session 9) were attributed to a higher overall taste (more acid) of L1T9. No significant differences in L1 at 0 and 6 days of storage were perceived (session 7).

Concerning the L2 sample, significant differences were detected when comparing the sample just produced (T0) with those at 6 (session 10) and 9 days of storage (session 11), while no differences were perceived when comparing L2T6 and L2T9 (session 12). In both significant sessions, a higher overall taste (more acid and sometimes unpleasant) of stored samples (L2T6 and L2T9) was perceived.

#### 3.5.3. Correlation between Sensory (QDA^®^) and Instrumental (Electronic Eye) Data

The data obtained from both sensory and instrumental approaches were statistically processed by MFA. The results showed that the sensory evaluation of the color intensity (QDA^®^) was correlated with the instrumental one (electronic eye). In particular, positive correlations were obtained between the sensory parameter and the instrumental variables (colors) that characterized the samples with darker coloring (Pearson’s correlation coefficients: 0.95 (variable 2165), 0.91 (variable 2712); *p* < 0.05), whereas negative correlations were found between the sensory parameter and the instrumental variables (colors) that described a lighter staining of the samples (Pearson correlation coefficients: 0.87 (variable 1878), 0.80 (variable 2696); *p* < 0.05).

## 4. Conclusions

This study demonstrated the efficacy of a powder formulation of a phenolic extract from olive vegetation water at improving the overall oxidative stability and sensory quality of raw and grilled beef hamburgers that had been previously subjected to cold storage for 9 days. The added phenolic compounds underwent a progressive decrease during the shelf-life period, but more than 55% and 63% of the added phenols were still retained (for L1 and L2, respectively) after 9 days of storage. On the other hand, cooking caused a more drastic reduction of phenolic compounds, leaving only 29.4% of the amount added in L1 at the beginning of storage. Both PE concentrations (87.5 and 175 mg of phenols/kg meat) proved to effectively reduce primary and secondary lipid oxidation, as well as cholesterol oxides, during the burgers’ shelf-life study and after cooking. In particular, PV, TBARs and total COPs were up to 1.4-, 4.5-, and 8.8-fold lower in PE raw hamburgers, respectively, than in the control samples; a similar trend was also noted in the cooked hamburgers (1,3-, 5.7-, and 4-fold lower). Moreover, the COR in the PE hamburgers was about half as much that of the control samples and never exceeded 0.5%.

Sensory analysis also confirmed the effectiveness of PE addition in beef hamburgers, having a positive effect especially on the intensity of the red color (raw product) as it resulted in a reduction of browning during storage. Furthermore, the presence of phenols was not perceived by panelists, so they did not negatively influence the organoleptic characteristics of the products. However, the discriminant test evidenced a qualitative decay of all products during storage, which was more relevant in the control sample. 

In conclusion, this study confirms that OMWW extracts rich in phenols could be an alternative for the reduction of synthetic additives in ground meat preparations, which would promote the formulation of healthier clean label products and improve the sustainability of the olive oil industry with a circular economy approach by further valorizing this olive processing by-product.

## Figures and Tables

**Table 1:** Proximate composition of raw hamburger samples after 0 and 9 days of storage. C, Control (minced beef meat + maltodextrin + starter cultures); L1, minced beef meat + starter cultures + 87.5 mg phenols/kg of meat; L2, minced beef meat + starter cultures + 175 mg phenols/kg of meat.

		Protein	Ash	Moisture	Fat
**Raw samples**	**(%)**				
	**0 days**				
	**C**	22.16 ± 1.14	1.74 ± 0.14	70.23 ± 0.65	5.87 ± 0.94
	**L1**	21.86 ± 1.27	1.68 ± 0.29	70.74 ± 0.80	5.73 ± 0.81
	**L2**	23.29 ± 1.15	1.85 ± 0.14	69.87 ± 0.60	4.99 ± 1.06
	**9 days**				
	**C**	22.65 ± 1.10	1.74 ± 0.06	70.16 ± 0.43	5.44 ± 0.79
	**L1**	22.46 ± 0.91	1.83 ± 0.05	70.42 ± 0.27	5.29 ± 0.92
	**L2**	22.19 ± 1.09	1.84 ± 0.12	70.31 ± 0.34	5.66 ± 0.99
	**Factor**	**F value**			
	**Form**	0.82 *ns*	1.64 *ns*	2.64 *ns*	0.37 *ns*
	**St**	0.00 *ns*	0.91 *ns*	0.01 *ns*	0.04 *ns*
	Form*St	2.17 *ns*	1.06 *ns*	1.49 *ns*	1.42 *ns*

Results as reported as mean ± s.d. of two independent replicates. No significant differences (Tukey’s test; *p* ≤ 0.05) between the same sample during the shelf-life, no significant differences (Tukey’s test; *p* ≤ 0.05) between treatments. Form, formulation; *ns*, non-significant; St, storage.

**Table 2:** Evolution of phenolic compounds of raw and grilled hamburger samples after 0, 6 and 9 days of storage. C, Control (minced beef meat + maltodextrine + starter cultures); L1, minced beef meat + starter cultures + 87.5 mg phenols/kg of meat; L2, minced beef meat + starter cultures + 175 mg phenols/kg of meat.

		3,4-DHPEA	*p*-HPEA	VB	3,4-DHPEA-EDA	Total Phenols
**Raw samples**	**(mg/Kg)**					
	**0 days**					
	**C**	-	-	-	-	-
	**L1**	29.37 ± 1.15 ^B,X^	4.46 ± 0.46 ^B,X^	10.30 ± 0.82 ^B,X^	40.83 ± 1,93 ^a,B,X^	84.96 ± 4.37 ^a,B,X^
	**L2**	40.98 ± 0.20 ^b,A,X^	9.23 ± 0.04 ^A,X^	22.80 ± 0.21 ^a,A,X^	95.29 ± 1.61 ^a,A,X^	168.29 ± 2.06 ^a,A,X^
	**6 days**					
	**C**	-	-	-	-	-
	**L1**	36.53 ± 1.92 ^B,X^	3.81 ± 0.10 ^B,X^	9.12 ± 0.53 ^B,X^	6.60 ± 0.16 ^b,B,X^	56.05 ± 2.71 ^b,B,X^
	**L2**	43.28 ± 0.56 ^b,A,X^	9.12 ± 0.02 ^A,X^	22.76 ± 0.10 ^a,A,X^	65.91 ± 3.00 ^b,A,X^	141.07 ± 3.69 ^b,A,X^
	**9 days**					
	**C**	-	-	-	-	-
	**L1**	32.08 ± 2.23 ^B,X^	3.32 ± 0.03 ^B,X^	9.79 ± 0.75 ^B,X^	2.82 ± 0.58 ^b,B,X^	48.01 ± 3.58 ^b,B,X^
	**L2**	48.87 ± 1.82 ^a,A,X^	8.82 ± 0.29 ^A,X^	20.80 ± 0.53 ^b,A,X^	28.37 ± 1.43 ^c,A,X^	106.86 ± 4.07 ^c,A,X^
**Grilled samples**	**0 days**					
	**C**	-	-	-	-	-
	**L1**	11.48 ± 0.87 ^a,B,Y^	3.35 ± 0.40 ^a,B,Y^	5.46 ± 0.49 ^B,Y^	*nd* ^Y^	20.29 ± 1.75 ^a,B,Y^
	**L2**	19.31 ± 0.90 ^a,A,Y^	8.14 ± 0.27 ^a,A,Y^	21.96 ± 0.65 ^a,A,Y^	*nd* ^Y^	49.42 ± 1.82 ^a,A,Y^
	**6 days**					
	**C**	-	-	-	-	-
	**L1**	4.56 ± 0.40 ^b,B,Y^	2.46 ± 0.28 ^ab,B,Y^	4.46 ± 0.17 ^B,Y^	*nd* ^Y^	11.48 ± 0.85 ^b,B,Y^
	**L2**	8.92 ± 1.29 ^b,A,Y^	7.27 ± 0.06 ^a,A,Y^	21.95 ± 0.87 ^a,A,Y^	*nd* ^Y^	38.14 ± 2.23 ^b,A,Y^
	**9 days**					
	**C**	-	-	-	-	-
	**L1**	*nd* ^c,B,Y^	1.38 ± 0.28 ^b,B,Y^	4.57 ± 0.17 ^B,Y^	*nd* ^Y^	5.95 ± 0.45 ^c,B,Y^
	**L2**	1.31 ± 0.30 ^c,A,Y^	4.66 ± 0.37 ^b,A,Y^	15.68 ± 0.68 ^b,A,Y^	*nd* ^Y^	21.65 ± 1.34 ^c,A,Y^
	**Factor**	**F value**				
	**Form**	2449.72 ***	4043.62 ***	6493.57 ***	3262.06 ***	4755.42 ***
	**St**	31.62 ***	89.59 ***	40.84 ***	944.93 ***	338.66 ***
	**Gr**	4031.08 ***	314.37 ***	244.21 ***	6484.39 ***	4794.17 ***
	Form*St	13.18 ***	24.79 ***	36.62 ***	320.75 ***	108.76 ***
	Form*Gr	1048.99 ***	91.57 ***	86.15 ***	3262.06 ***	1613.62 ***
	St*Gr	146.39 ***	29.44 ***	12.08 ***	944.93 ***	55.62 ***
	Form*St*Gr	50.22 ***	14.75 ***	8.84 ***	320.75 ***	17.07 ***

Results as reported as means ± s.d. of 2 independent replicates. a–c indicate significant differences (Tukey’s test; *p* ≤ 0.05) within the same sample during the shelf-life. A–B indicate significant differences (Tukey’s test; *p* ≤ 0.05) among treatments, X–Y indicate significant differences (Tukey’s test; *p* ≤ 0.05) between raw and grilled samples. *** *p* < 0.001. 3,4-DHPEA, hydroxytyrosol; 3,4-DHPEA-EDA, oleacein; Form, formulation; Gr, grilling; *nd*, not determined; *p*-HPEA, tyrosol; St, storage; VB, verbascoside.

**Table 3:** Lipid content and main lipid classes profile of raw and grilled hamburger samples after 0, 6 and 9 days of storage. C, Control (minced beef meat + maltodextrine + starter cultures); L1, minced beef meat + starter cultures + 87.5 mg phenols/kg of meat; L2, minced beef meat + starter cultures + 175 mg phenols/kg of meat.

		Lipid Content	FFA	MAG	STE	DAG	E-STE	TAG
		(%)	(% of Total Lipids)					
**Raw samples**	**0 days**							
	**C**	4.93 ± 0.60 ^Y^	5.87 ± 0.21 ^X^	0.26 ± 0.01 ^X^	1.87 ± 0.44	4.26 ± 0.79	2.01 ± 0.05	85.62 ± 1.18
	**L1**	6.33 ± 0.53	4.15 ± 0.70	0.13 ± 0.01	1.10 ± 0.12	3.50 ± 0.50 ^Y^	1.18 ± 0.64 ^Y^	89.87 ± 1.01 ^X^
	**L2**	5.29 ± 0.45 ^Y^	3.51 ± 0.45	0.10 ± 0.02	1.42 ± 0.38	4.20 ± 0.59	2.12 ± 0.07	88.58 ± 1.25
	**6 days**							
	**C**	4.98 ± 0.30 ^Y^	6.12 ± 0.54	0.24 ± 0.03 ^X^	1.88 ± 0.45	4.18 ± 1.01	1.68 ± 0.57	85.80 ± 1.45
	**L1**	5.13 ± 1.02 ^Y^	5.50 ± 1.28	0.16 ± 0.06	1.28 ± 0.36	4.49 ± 0.76	2.24 ± 0.12	86.26 ± 1.91
	**L2**	5.39 ± 0.62 ^Y^	3.10 ± 0.21 ^Y^	0.08 ± 0.02	1.35 ± 0.15	4.51 ± 0.54	2.41 ± 0.26	88.49 ± 0.46 ^X^
	**9 days**							
	**C**	5.04 ± 0.35 ^Y^	5.55 ± 0.12	0.18 ± 0.01	1.70 ± 0.06	3.83 ± 0.12	2.00 ± 0.02	86.68 ± 0.17
	**L1**	5.29 ± 0.45 ^Y^	4.36 ± 0.74	0.11 ± 0.00	1.14 ± 0.16	4.34 ± 0.66	2.19 ± 0.02	87.79 ± 0.07
	**L2**	6.06 ± 0.18 ^Y^	5.13 ± 0.56 ^X^	0.15 ± 0.01 ^X^	1.13 ± 0.15	4.64 ± 0.24	2.28 ± 0.01 ^X^	86.61 ± 0.76
**Grilled samples**	**0 days**							
	**C**	6.12 ± 0.47 ^X^	5.38 ± 0.21 ^Y^	0.19 ± 0.02 ^Y^	1.97 ± 0.22	4.81 ± 0.63	2.00 ± 0.07	85.57 ± 0.91
	**L1**	6.15 ± 0.22	3.56 ± 0.55	0.10 ± 0.03	1.66 ± 0.27	4.84 ± 0.33 ^X^	2.20 ± 0.19 ^X^	87.57 ± 0.57 ^Y^
	**L2**	6.21 ± 0.10 ^X^	4.21 ± 0.39	0.13 ± 0.02	1.56 ± 0.10	5.97 ± 0.26	2.11 ± 0.23	86.86 ± 0.63
	**6 days**							
	**C**	7.09 ± 0.35 ^X^	6.06 ± 0.37	0.18 ± 0.01 ^Y^	1.65 ± 0.10	4.29 ± 0.20	1.95 ± 0.31	85.79 ± 0.28
	**L1**	6.47 ± 0.90 ^X^	4.98 ± 0.32	0.10 ± 0.04	1.66 ± 0.38	5.98 ± 0.88	2.13 ± 0.12	87.08 ± 1.42
	**L2**	6.66 ± 1.15 ^X^	4.07 ± 0.24 ^X^	0.11 ± 0.01	1.69 ± 0.22	5.24 ± 0.51	2.18 ± 0.08	86.75 ± 0.88 ^Y^
	**9 days**							
	**C**	7.49 ± 1.02 ^X^	5.24 ± 1.03	0.17 ± 0.04	1.72 ± 0.60	4.37 ± 0.99	1.79 ± 0.57	86.65 ± 2.00
	**L1**	6.47 ± 0.74 ^X^	3.76 ± 0.32	0.10 ± 0.01	1.67 ± 0.33	4.87 ± 0.46	2.06 ± 0.08	87.42 ± 0.51
	**L2**	6.89 ± 1.22 ^X^	3.85 ± 0.24 ^Y^	0.10 ± 0.01 ^Y^	1.44 ± 0.33	4.45 ± 0.52	2.09 ± 0.06 ^Y^	88.02 ± 1.16
	**Factor**	**F value**						
	**Form**	0.30 *ns*	46.62 ***	58.68 ***	8.30 **	1.32 *ns*	3.66 *	11.52 ***
	**St**	1.05 *ns*	2.34 *ns*	1.15 *ns*	0.82 *ns*	0.52 *ns*	0.53 *ns*	1.81 *ns*
	**Gr**	35.04 ***	3.59 *ns*	11.10 **	5.90 *	6.89 *	0.01 *ns*	1.35 *ns*
	Form*St	0.72 *ns*	4.03 **	3.21 *	0.35 *ns*	0.74 *ns*	1.56 *ns*	1.78 *ns*
	Form*Gr	2.03 *ns*	3.07 *ns*	4.01 *	3.68 *	0.77 *ns*	1.74 *ns*	0.84 *ns*
	St*Gr	2.35 *ns*	2.05 *ns*	0.24 *ns*	0.21 *ns*	0.33 *ns*	2.14 *ns*	1.78 *ns*
	Form*St*Gr	0.34 *ns*	3.45 *	4.58 **	0.08 *ns*	0.84 *ns*	1.91 *ns*	2.52 *ns*

Results as reported as means ± s.d. of three independent replicates. X−Y indicate significant differences (Tukey’s test; *p* ≤ 0.05) between raw and grilled samples. * *p* < 0.05, ** *p* < 0.01, *** *p* < 0.001. DAG, diacylglycerols; E-STE, esterified sterols; FFA, free fatty acids; Form, formulation; Gr, grilling; MAG, monoacylglycerols; *ns,* non-significant; STE, sterols; St, storage; TAG, triacylglycerols.

**Table 4:** Fatty acid classes (expressed as % of total fatty acids) of raw and grilled hamburgers after 0, 6 and 9 days of storage. C, Control (minced beef meat + maltodextrine + starter cultures); L1, minced beef meat + starter cultures + 87.5 mg phenols/kg of meat; L2, minced beef meat + starter cultures + 175 mg phenols/kg of meat.

		SFA	MUFA	PUFA	*n*-3	*n*-6
**Raw samples**	**(% Total Fatty Acids)**					
	**0 days**					
	**C**	38.95 ± 1.22	57.40 ± 1.13	3.65 ± 0.14	0.68 ± 0.10	2.39 ± 0.21
	**L1**	40.13 ± 0.56	55.81 ± 0.50	4.06 ± 0.55 ^a^	0.56 ± 0.18	2.80 ± 0.46
	**L2**	38.42 ± 1.42	57.56 ± 0.39	4.01 ± 1.26	0.71 ± 0.16	2.50 ± 0.90
	**6 days**					
	**C**	36.90 ± 3.79 ^B^	58.94 ± 3.09	4.17 ± 0.95	0.65 ± 0.15	2.89 ± 0.82 ^A^
	**L1**	40.59 ± 1.59 ^A^	57.58 ± 1.29	4.82 ± 0.52 ^a^	0.73 ± 0.06	2.99 ± 0.48 ^B^
	**L2**	40.01 ± 3.16 ^B^	57.71 ± 2.60	4.27 ± 0.58	0.58 ± 0.03	2.43 ± 0.40 ^AB,Y^
	**9 days**					
	**C**	39.35 ± 1.36	56.67 ± 1.64	3.98 ± 0.37	0.61 ± 0.07	2.66 ± 0.44
	**L1**	41.67 ± 0.67	56.84 ± 0.85	3.50 ± 0.19 ^b,Y^	0.65 ± 0.17	2.77 ± 0.30 ^Y^
	**L2**	38.30 ± 0.85 ^Y^	58.64 ± 1.49 ^Y^	3.07 ± 1.49	0.53 ± 0.01 ^X^	2.01 ± 1.10
**Grilled samples**	**0 days**					
	**C**	39.70 ± 1.32	56.37 ± 0.99	3.93 ± 0.95	0.64 ± 0.16	2.81 ± 0.77
	**L1**	42.49 ± 3.78	55.12 ± 1.78	2.27 ± 2.32	0.51 ± 0.26	2.70 ± 1.77
	**L2**	39.26 ± 0.43 ^b^	57.44 ± 0.91	2.82 ± 0.49	0.52 ± 0.08	2.12 ± 0.49
	**6 days**					
	**C**	39.55 ± 2.51	56.87 ± 1.97	3.58 ± 0.86	0.48 ± 0.05	2.78 ± 0.75
	**L1**	40.10 ± 1.92	55.10 ± 3.34	3.12 ± 0.95	0.62 ± 0.21	2.19 ± 0.62
	**L2**	39.73 ± 0.53 ^b^	57.33 ± 0.63	2.94 ± 0.14	0.43 ± 0.11	2.32 ± 0.23 ^X^
	**9 days**					
	**C**	37.80 ± 4.39	57.83 ± 3.67	4.00 ± 0.16 ^A^	0.59 ± 0.18	2.99 ± 0.28
	**L1**	39.70 ± 2.54	56.99 ± 2.09	3.31 ± 0.55 ^AB,X^	0.57 ± 0.23	2.40 ± 0.15 ^X^
	**L2**	41.18 ± 0.21 ^a,X^	56.14 ± 0.27 ^X^	2.69 ±0.38 ^B^	0.41 ± 0.04 ^Y^	2.17 ± 0.42
	**Factor**	**F value**				
	**Form**	2.96 *ns*	1.19 *ns*	10.08 ***	1.70 *ns*	10.16 ***
	**St**	0.04 *ns*	0.40 *ns*	1.88 *ns*	0.07 *ns*	1.28 *ns*
	**Gr**	0.39 *ns*	2.37 *ns*	0.35 *ns*	7.20 *	4.94 *
	Form*St	0.20 *ns*	0.18 *ns*	0.50 *ns*	2.16 *ns*	0.64 *ns*
	Form*Gr	0.29 *ns*	0.10 *ns*	0.36 *ns*	0.93 *ns*	0.21 *ns*
	St*Gr	0.24 *ns*	0.56 *ns*	1.80 *ns*	0.20 *ns*	1.89 *ns*
	Form*St*Gr	1.61 *ns*	0.88 *ns*	4.48 **	1.09 *ns*	3.32 *

Results as reported as means ± s.d. of three independent replicates. a–b indicate significant differences (Tukey’s test; *p* ≤ 0.05) between the same sample during the shelf-life, A–B indicate significant differences (Tukey’s test; *p* ≤ 0.05) between treatments, X–Y indicate significant differences (Tukey’s test; *p* ≤ 0.05) between raw and grilled samples. * *p* < 0.05, ** *p* < 0.01, *** *p* < 0.001. Form, formulation; Gr, grilling; MUFA, monounsaturated fatty acids; *ns,* non-significant; PUFA, polyunsaturated fatty acids; SFA, saturated fatty acids; St, storage; UFA, unsaturated fatty acids.

**Table 5:** Ratios of fatty acid classes, atherogenic index (AI) and thrombogenic index (TI) of raw and grilled hamburgers after 0, 6, and 9 days of storage. C, control (minced beef meat + maltodextrine + starter cultures); L1, minced beef meat + starter cultures + 87.5 mg phenols/kg of meat; L2, minced beef meat + starter cultures + 175 mg phenols/kg of meat.

		*n*-6/*n*-3	UFA/SFA	PUFA/SFA	AI	TI
**Raw samples**	**0 days**					
	**C**	3.59 ± 0.80	1.57 ± 0.08	0.09 ± 0.01	0.58 ± 0.01	1.17 ± 0.06
	**L1**	5.45 ± 2.00 ^a^	1.49 ± 0.03	0.10 ± 0.01 ^a^	0.60 ± 0.01	1.24 ± 0.04
	**L2**	3.69 ± 1.80	1.60 ± 0.09	0.11 ± 0.04	0.56 ± 0.03	1.14 ± 0.07
	**6 days**					
	**C**	4.59 ± 1.44 ^A^	1.73 ± 0.29	0.12 ± 0.04 ^A^	0.53 ± 0.08	1.08 ± 0.18
	**L1**	4.09 ± 0.81 ^b,B^	1.47 ± 0.09	0.05 ± 0.01 ^b,B^	0.63 ± 0.04	1.22 ± 0.05
	**L2**	4.18 ± 0.68 ^AB,Y^	1.51 ± 0.19	0.06 ± 0.02 ^AB^	0.62 ± 0.06	1.22 ± 0.15
	**9 days**					
	**C**	4.42 ± 1.17	1.54 ± 0.09	0.10 ± 0.01	0.58 ± 0.02	1.19 ± 0.07
	**L1**	4.26 ± 0.85 ^b,Y^	1.40 ± 0.04	0.04 ± 0.00 ^b^	0.66 ± 0.01	1.27 ± 0.06
	**L2**	3.77 ± 2.04	1.61 ± 0.06 ^X^	0.08 ± 0.04	0.57 ± 0.02 ^Y^	1.15 ± 0.04 ^Y^
**Grilled samples**	**0 days**					
	**C**	4.58 ± 1.66 ^B^	1.52 ± 0.08	0.10 ± 0.03	0.61 ± 0.04	1.21 ± 0.08
	**L1**	5.29 ± 1.92 ^A^	1.36 ± 0.21	0.06 ± 0.06	0.69 ± 0.08	1.41 ± 0.23
	**L2**	4.14 ± 1.42 ^B^	1.53 ± 0.03 ^a^	0.07 ± 0.01	0.59 ± 0.03	1.20 ± 0.02 ^b^
	**6 days**					
	**C**	5.85 ± 1.84	1.54 ± 0.17	0.09 ± 0.03	0.60 ± 0.06	1.21 ± 0.13
	**L1**	3.99 ± 0.28	1.46 ± 0.17	0.08 ± 0.03	0.58 ± 0.05	1.23 ± 0.11
	**L2**	5.71 ± 1.73 ^X^	1.52 ± 0.03 ^a^	0.07 ± 0.00	0.59 ± 0.02	1.22 ± 0.22 ^b^
	**9 days**					
	**C**	5.42 ± 1.80	1.66 ± 0.31	0.11 ± 0.02 ^A^	0.56 ± 0.07	1.13 ± 0.20
	**L1**	4.85 ± 2.38 ^X^	1.53 ± 0.16	0.08 ± 0.02 ^AB^	0.61 ± 0.06	1.22 ± 0.15
	**L2**	5.42 ± 1.40	1.43 ± 0.01 ^b,Y^	0.07 ± 0.01 ^B^	0.63 ± 0.03 ^X^	1.30 ± 0.01 ^a,X^
	**Factor**	**F value**				
	**Form**	1.37 *ns*	3.23 *ns*	9.46 ***	4.01 *	2.30 *ns*
	**St**	0.98 *ns*	0.03 *ns*	1.31 *ns*	0.18 *ns*	0.11 *ns*
	**Gr**	14.99 ***	0.57 *ns*	0.14 *ns*	0.14 *ns*	1.42 *ns*
	Form*St	2.68 *	0.24 *ns*	0.56 *ns*	0.69 *ns*	0.20 *ns*
	Form*Gr	0.93 *ns*	0.26 *ns*	0.45 *ns*	0.31 *ns*	0.18 *ns*
	St*Gr	0.48 *ns*	0.39 *ns*	1.36 *ns*	0.48 *ns*	0.18 *ns*
	Form*St*Gr	0.32 *ns*	1.47 *ns*	3.92 *	2.73 *	1.43 *ns*

Results as reported as means± s.d. of three independent replicates. a–b indicate significant differences (Tukey’s test; *p* ≤ 0.05) between the same sample during the shelf-life, A–B indicate significant differences (Tukey’s test; *p* ≤ 0.05) between treatments, X–Y indicate significant differences (Tukey’s test; *p* ≤ 0.05) between raw and grilled samples. * *p* < 0.05, *** *p* < 0.001. AI, atherogenic index; Form, formulation; Gr, grilling; *ns,* non-significant; PUFA, polyunsaturated fatty acids; SFA, saturated fatty acids, St, storage; TI, thrombogenic index; UFA, unsaturated fatty acids.

**Table 6:** PV, TBARs, cholesterol, total COPs, and COR of raw and grilled hamburgers after 0, 6, and 9 days of storage. C, control (minced beef meat + maltodextrine + starter cultures); L1, minced beef meat + starter cultures + 87.5 mg phenols/kg of meat; L2, minced beef meat + starter cultures + 175 mg phenols/kg of meat.

		PV	TBARs	Cholesterol	Total COPs	COR
		(meq O_2_/kg Fat)	(mg MDA/kg Burger)	(mg/kg Burger)		(%)
**Raw samples**	**0 days**					
	**C**	0.91 ± 0.08 ^c,Y^	0.81 ± 0.16 ^c,A^	538.27 ± 20.32 ^Y^	1.59 ± 0.30 ^b,Y^	0.29 ± 0.05 ^b,A^
	**L1**	0.89 ± 0.09 ^c,Y^	0.53 ± 0.04 ^b,AB^	743.04 ± 66.67	1.28 ± 0.14 ^Y^	0.17 ± 0.04 ^B,Y^
	**L2**	0.84 ± 0.06 ^b,Y^	0.48 ± 0.06 ^b,B^	728.32 ± 17.02 ^Y^	0.90 ± 0.25 ^Y^	0.12 ± 0.03 ^B,Y^
	**6 days**					
	**C**	3.57 ± 0.17 ^b,Y^	4.19 ± 0.20 ^b,A,X^	560.44 ± 22.92 ^Y^	5.38 ± 0.07 ^a,A,Y^	0.97 ± 0.17 ^a,A^
	**L1**	3.01 ± 0.20 ^b,Y^	1.07 ± 0.31 ^ab,B^	773.74 ± 8.23 ^Y^	2.03 ± 0.35 ^B,Y^	0.26 ± 0.04 ^B,Y^
	**L2**	2.88 ± 0.15 ^a,Y^	0.55 ± 0.09 ^a,C^	827.74 ± 79.71 ^Y^	1.17 ± 0.36 ^B,Y^	0.14 ± 0.06 ^B,Y^
	**9 days**					
	**C**	5.63 ± 0.20 ^a,A,Y^	4.86 ± 0.57 ^a,A^	587.53 ± 63.62 ^Y^	6.32 ± 0.71 ^a,A,Y^	1.08 ± 0.01 ^a,A^
	**L1**	4.05 ± 0.31 ^a,B,Y^	1.22 ± 0.25 ^a,B^	778.46 ± 25.49 ^Y^	2.31 ± 0.48 ^B^	0.30 ± 0.04 ^B^
	**L2**	3.58 ± 0.38 ^a,B,Y^	0.51 ± 0.03 ^a,C^	926.07 ± 51.88 ^Y^	1.19 ± 0.28 ^B,Y^	0.13 ± 0.04 ^B,Y^
**Grilled sample**	**0 days**					
	**C**	8.04 ± 0.68 ^c,X^	0.93 ± 0.14 ^c,A^	1040.97 ± 113.46 ^X^	2.88 ± 0.42 ^c,X^	0.30 ± 0.07 ^c^
	**L1**	6.14 ± 0.55 ^b,X^	0.63 ± 0.09 ^B^	1145.94 ± 166.70	3.98 ± 0.21 ^X^	0.35 ± 0.06 ^X^
	**L2**	5.65 ± 0.66 ^c,X^	0.52 ± 0.05 ^b,C^	1097.02 ± 59.73 ^X^	2.85 ± 0.24 ^X^	0.26 ± 0.04 ^X^
	**6 days**					
	**C**	13.62 ± 0.92 ^b,A,X^	3.17 ± 0.47 b^A,Y^	1146.78 ± 124.46 ^X^	11.88 ± 0.03 ^b,A,X^	1.07 ± 0.30 ^b,A^
	**L1**	11.44 ± 0.86 ^a,A,X^	0.97 ± 0.02 ^B^	1022.39 ± 9.76 ^X^	4.96 ± 1.01 ^B,X^	0.49 ± 0.10 ^B,X^
	**L2**	9.15 ± 0.72 ^b,B,X^	0.64 ± 0.04 ^a,B^	1041.24 ± 10.81 ^X^	3.04 ± 0.51 ^B,X^	0.29 ± 0.05 ^B,X^
	**9 days**					
	**C**	17.74 ± 1.01 ^a,A,X^	4.07 ± 0.20 ^a,A^	1126.62 ± 100.13 ^X^	17.00 ± 1.47 ^a,A,X^	1.51 ± 0.01 ^a,A,X^
	**L1**	14.87 ± 1.09 ^a,AB,X^	1.02 ± 0.16 ^B^	1225.14 ± 100.72 ^X^	3.70 ± 0.30 ^B^	0.31 ± 0.10 ^B^
	**L2**	13.12 ± 0.99 ^a,B,X^	0.72 ± 0.03 ^a,B^	1104.97 ± 114.31 ^X^	3.22 ± 0.76 ^B,X^	0.30 ± 0.10 ^B,X^
	**Factor**	**F value**				
	**Form**	28.13 ***	90.43 ***	2.44 *ns*	705.66 ***	284.00 ***
	**St**	127.38 ***	35.94 ***	0.88 *ns*	238.65 ***	85.21 ***
	**Gr**	769.06 ***	4.26 *ns*	105.30 ***	742.57 ***	49.33 ***
	Form*St	0.86 *ns*	17.17 ***	0.50 *ns*	175.40 ***	60.26 ***
	Form*Gr	5.05 *ns*	1.10 *ns*	3.50 *ns*	110.80 ***	0.87 *ns*
	St*Gr	29.68 ***	1.26 *ns*	0.01 *ns*	42.23 ***	0.74 *ns*
	Form*St*Gr	0.73 *ns*	0.94 *ns*	1.72 *ns*	58.73 ***	5.78 **

Results as reported as means ± s.d. of three independent replicates. a–c indicate significant differences (Tukey’s test; *p* ≤ 0.05) between the same samples during the shelf-life, A–C indicate significant differences (Tukey’s test; *p* ≤ 0.05) between treatments, X–Y indicate significant differences (Tukey’s test; *p* ≤ 0.05) between raw and grilled samples. ** *p* < 0.01, *** *p* < 0.001. COR, cholesterol oxidation ratio; COPs, cholesterol oxidation products; Form, formulation; Gr, grilling; *ns,* non-significant; PV, peroxide value; St, storage; TBARs, thiobarbituric acid reactive substances.

**Table 7:** Mean values (five replicates) of the intensity of the attributes evaluated by QDA^®^ of burger samples (C, Control (minced beef meat + maltodextrine + starter cultures); L1, minced beef meat + starter cultures + 87.5 mg phenols/kg of meat; L2, minced beef meat + starter cultures + 175 phenols/kg of meat) at all of the storage times (T0, T6, and T9), expressed on a scale from 0 to 100 (0 indicates the absence of perception of the attribute, while 100 corresponds to the maximum perception of the attribute) * extremes of the scale.

Sensory Descriptors								
Sample Codes	Beef Flavor	Bloody	Salty	Juiciness	Granularity	Tenderness	Fat/Connective Tissue	Color
	(low; high) *	(low; high) *	(low; high) *	(low; high) *	(low; high) *	(low; high) *	(low; high)*	(red; brown) *
CT0	47.2 ^ab^	26.5 ^ab^	38.2 ^b^	55.3 ^a^	36.7 ^a^	55.6 ^a^	42.3 ^a^	67.2 ^bc^
CT6	39.1 ^bc^	28.7 ^a^	39.9 ^ab^	52.3 ^a^	32.9 ^a^	54.8 ^a^	43.1 ^a^	77.9 ^ab^
CT9	37.7 ^c^	24.5 ^a-c^	38.9 ^b^	37.6 ^d^	38.6 ^a^	45.3 ^ab^	49.6 ^a^	78.6 ^a^
L1T0	51.9 ^a^	19.0 ^bc^	41.5 ^ab^	51.3 ^ab^	29.8 ^a^	55.7 ^a^	39.3 ^a^	42.0 ^ef^
L1T6	50.2 ^a^	19.3 ^bc^	41.4 ^ab^	51.3 ^ab^	38.3 ^a^	51.1 ^ab^	40.8 ^a^	47.3 ^d–f^
L1T9	50.8 ^a^	24.1 ^a–c^	42.0 ^ab^	39.1 ^cd^	38.2 ^a^	41.6 ^b^	40.2 ^a^	49.9 ^de^
L2T0	52.0 ^a^	22.6 ^a–c^	40.4 ^ab^	51.3 ^ab^	34.6 ^a^	50.6 ^ab^	43.6 ^a^	38.0 ^f^
L2T6	54.9 ^a^	20.1 ^bc^	42.6 ^ab^	49.8 ^a–c^	33.9 ^a^	54.2 ^a^	48.7 ^a^	39.7 ^ef^
L2T9	48.6 ^a^	17.5 ^c^	45.9 ^a^	40.0 ^b–d^	36.7 ^a^	39.5 ^b^	48.4 ^a^	56.4 ^cd^

Different letters (a–f) indicate significantly different values from each other (multiple comparison test, Fisher’s LDS with *p* < 0.05). * sensation intensity of attributes.

**Table 8:** Sessions, compared samples, number of subjects involved, number of correct given answers and significance level of the triangle test conducted on hamburger samples (C, Control (minced beef meat + maltodextrine + starter cultures); L1, minced beef meat + starter cultures + 87.5 mg phenols/kg of meat; L2, minced beef meat + starter cultures + 175 phenols/kg of meat) at all the storage times (T0, T6, and T9).

Session n.	Compared Samples	Judges n.	Correct Answers	Significance
1	CT0 vs. L1T0	30	15	0.05
2	CT0 vs. L2T0	30	17	0.01
3	L1T0 vs. L2T0	30	14	0.1
4	CT0 vs. CT6	30	16	0.05–0.01
5	CT0 vs. CT9	30	13	0.2
6	CT6 vs. CT9	30	9	*ns*
7	L1T0 vs. L1T6	28	10	*ns*
8	L1T0 vs. L1T9	28	13	0.2
9	L1T6 vs. L1T9	28	16	0.01
10	L2T0 vs. L2T6	28	12	0.3
11	L2T0 vs. L2T9	28	13	0.2
12	L2T6 vs. L2T9	28	10	*ns*

The significance is expressed in terms of α-risk level. *ns* indicates no significant perceptible difference between samples was found.

## Data Availability

The data presented in this study are available in this manuscript.

## Supplementary Materials

The following are available online at https://www.mdpi.com/article/10.3390/antiox10121969/s1. Table S1: Total fatty acid composition (expressed as % of total fatty acid) of raw and grilled beef burger after 0, 6, and 9 days of storage. C, Control samples (minced beef+ maltodextrin+ starter cultures); L1, minced beef+ starter cultures + 87.5 mg of phenols/kg of meat; L2, minced beef+ starter cultures + 175 mg of phenols/kg of meat.
