# Supplementary material for: Improved Oxidative Stability and Sensory Quality of Beef Hamburgers Enriched with a Phenolic Extract from Olive Vegetation Water
Source: Antioxidants (Basel). 2021 Dec 9;10(12):1969. doi: 10.3390/antiox10121969 (PMC8750197; doi:10.3390/antiox10121969)
Supplement: Supplementary file 1 [file antioxidants-10-01969-s001.zip › antioxidants-1468328-supplementary.pdf]

Table S1. Total Fatty acid composition (expressed as % of total fatty acid) of raw and grilled beef burger after 0,6 and 9 days of storage. C, Control samples (minced beef+ maltodextrin+ starter cultures); L1, minced beef+ starter cultures+87.5 mg of phenols / Kg of meat; L2, minced beef+ starter cultures+175 mg of phenols / Kg of meat.

		C12:0	C14:0	C14:1	C15:0	C15:1	C16:0	C16:1*t n7*	C16:1 *n7*
		(% Total Fatty Acid)							
Raw samples		0 days							
	C	0.06±0.00[Y]	2.66±0.19	1.03±0.17	0.35±0.05	0.17±0.03	24.60±0.74	6.39±0.86[X]	0.42±0.04[X]
	L1	0.08±0.02	2.86±0.30	0.87±0.11	0.40±0.07	0.17±0.00	24.15±0.72	5.62±0.59[X]	0.24±0.15[X]
	L2	0.06±0.01	2.57±0.13	0.98±0.22	0.35±0.03	0.18±0.01	23.86±1.99	5.88±1.26[X]	0.37±0.05[X]
		6 days							
	C	0.08±0.02	2.60±0.30	0.93±0.09	0.37±0.07	0.17±0.01	22.70±2.55	6.12±0.68[X]	0.11±0.03[X]
	L1	0.08±0.02	2.89±0.43	1.14±0.18	0.40±0.10	0.19±0.01	25.85±0.50	6.96±0.85[X]	0.26±0.19[X]
	L2	0.07±0.02	2.90±0.35	1.08±0.26	0.39±0.09	0.20±0.00[A]	24.93±0.47	5.76±0.61[X]	0.39±0.03[X]
		9 days							
	C	0.07±0.01	2.74±0.17	0.90±0.24	0.41±0.05	0.18±0.02[B]	23.88±1.06	5.89±0.96[X]	0.11±0.04[Y]
	L1	0.07±0.01	3.01±0.14	1.12±0.24	0.41±0.04	0.19±0.02[AB]	26.21±1.12[a]	6.63±1.05[X]	0.38±0.02[Y]
	L2	0.07±0.01	2.68±0.15	1.14±0.29	0.36±0.06	0.19±0.02	24.22±1.75	6.95±1.34[X]	0.36±0.05[Y]
Grilled samples		0 days							
	C	0.09±0.00[X]	2.84±0.15[AB]	1.18±0.27	0.38±0.04	0.18±0.01	25.14±1.83	4.79±0.77[X]	0.10±0.02[Y]
	L1	0.09±0.02	3.20±0.29[A]	1.05±0.18	0.43±0.06	0.19±0.01	26.18±2.23	4.42±0.53[X]	0.07±0.03[Y]
	L2	0.07±0.01	2.58±0.13[B]	1.89±0.99	0.34±0.00	0.18±0.01	24.89±1.35	4.83±0.85[X]	0.09±0.01[Y]
		6 days							
	C	0.08±0.01	2.92±0.08	1.03±0.18	0.40±0.02	0.18±0.01	24.26±2.22	4.38±0.51[X]	0.08±0.02[Y]
	L1	0.07±0.01	2.13±1.30	0.89±0.39	0.39±0.05	0.19±0.01	25.01±0.58	4.57±0.54[X]	0.08±0.01[Y]
	L2	0.07±0.01	2.67±0.18	1.07±0.20	0.39±0.04	0.18±0.02	24.80±1.26	4.59±0.63[X]	0.08±0.01[Y]
		9 days							
	C	0.08±0.01	2.72±0.39[a]	1.04±0.28	0.39±0.07	0.18±0.00	23.16±0.88	4.10±0.28[X]	0.08±0.01[Y]
	L1	0.08±0.02	2.82±0.49[ab]	1.11±0.13	0.39±0.08	0.19±0.01	25.15±0.67	4.66±0.47[X]	0.09±0.01[Y]
	L2	0.08±0.01	2.96±0.04[a]	1.00±0.25	0.41±0.04	0.20±0.02	25.04±1.73	4.27±0.65[X]	0.09±0.03[Y]
	**Factor**	**F value**							
	**Form**	0.71 *ns*	0.07 *ns*	1.85 *ns*	0.51 *ns*	2.79 *ns*	5.04 *ns*	3.16 *ns*	0.05 *ns*
	**St**	0.05 *ns*	0.50 *ns*	1.83 *ns*	0.77 *ns*	1.02 *ns*	0.00 *ns*	0.99 *ns*	0.83 *ns*
	**Gr**	2.51 *ns*	0.10 *ns*	2.47 *ns*	0.22 *ns*	0.00 *ns*	0.12 *ns*	0.76***	312.06***
	**Form*St**	0.20 *ns*	1.00 *ns*	1.06 *ns*	0.22 *ns*	0.68 *ns*	0.93 *ns*	0.69 *ns*	0.78 *ns*

**Form*Gr**	0.17 *ns*	0.84 *ns*	1.77*	0.07 *ns*	0.21 *ns*	0.17 *ns*	4.10 *ns*	0.65 *ns*
**St*Gr**	1.52 *ns*	0.74 *ns*	3.47 *ns*	0.02 *ns*	0.42 *ns*	0.39 *ns*	2.10 *ns*	0.32 *ns*
**Form*St*Gr**	0.19 *ns*	1.29 *ns*	2.46 *ns*	0.52 *ns*	0.65 *ns*	1.67 *ns*	0.60 *ns*	0.43 *ns*

Results as reported like means± standard deviation. a–b indicates significant differences ($p < 0.05$) between the same sample during the shelf-life, A-C indicate significant differences ($p < 0.05$) between treatments, X-Y indicate significant differences (Tukey's test; $p \leq 0.05$) between raw and grilled samples. *$p < 0.05$, **$p < 0.01$, ***$p < 0.001$. Form, formulation; Gr, grilling; St, storage.

		C17:0	C17:1	C18:0	C18:1 *n9*	C18:1 *cis11*	C18:2	C20:0	C18:3 *n3*
		**(% Total Fatty Acid)**							
**Raw samples**	**0 days**								
	**C**	0.73±0.15	0.80±0.03	10.33±1.21	47.56±0.56	0.53±0.14	2.39±0.21	0.07±0.01	0.25±0.10
	**L1**	0.75±0.12	0.76±0.08	11.64±0.72	47.34±0.43	0.35±0.03	2.80±0.46	0.08±0.01	0.20±0.11
	**L2**	0.69±0.09	0.77±0.06	10.63±1.23	48.14±0.39	0.52±0.18	2.50±0.90	0.08±0.01	0.24±0.10
	**6 days**								
	**C**	0.71±0.11	0.82±0.09	10.21±1.76	49.78±3.09	0.53±0.18	2.89±0.82	0.08±0.01	0.18±0.08
	**L1**	0.74±0.16	0.84±0.04	10.55±1.31	47.51±0.30	0.36±0.13	2.99±0.48	0.06±0.00	0.12±0.08
	**L2**	0.78±0.20	0.79±0.08	10.82±2.37	47.74±1.00	0.57±0.20	2.43±0.40	0.07±0.01	0.16±0.10
	**9 days**								
	**C**	0.78±0.15	0.82±0.04	11.23±1.72	48.03±0.83	0.36±0.13	2.66±0.44	0.08±0.02	0.20±0.10
	**L1**	0.80±0.12	0.82±0.03	11.05±1.51	47.12±0.56	0.35±0.10	2.77±0.30	0.12±0.09	0.10±0.10
	**L2**	0.69±0.11	0.80±0.05	10.10±1.11	48.10±0.64	0.66±0.24	2.01±1.10	0.07±0.02	0.18±0.02
**Grilled samples**	**0 days**								
	**C**	0.71±0.11	0.83±0.02	10.29±1.36	47.88±0.77	0.55±0.15	2.81±0.77	0.07±0.01	0.25±0.10
	**L1**	0.83±0.13	0.84±0.08	11.43±1.75	47.50±1.43	0.37±0.09	2.70±0.77	0.07±0.01	0.29±0.07
	**L2**	0.67±0.13	0.84±0.03	10.53±1.43	48.30±0.73	0.45±0.20	2.12±0.49	0.07±0.01	0.17±0.06
	**6 days**								
	**C**	0.77±0.08	0.85±0.04	10.89±1.24	49.01±1.99	0.40±0.05	2.78±0.75	0.07±0.01	0.19±0.06
	**L1**	0.81±0.27	0.86±0.02	10.77±1.70	47.28±2.84	0.41±0.13	2.19±0.62	0.08±1.28	0.39±0.30
	**L2**	0.74±0.13	0.86±0.06	10.85±1.22	49.19±0.76	0.44±0.10	2.32±0.23	0.08±0.00	0.21±0.08
	**9 days**								
	**C**	0.73±0.18	0.85±0.03	10.46±2.87	49.94±2.94	0.60±0.23	2.99±0.28	0.07±0.02	0.24±0.14
	**L1**	0.73±0.15	0.85±0.05	10.32±1.44	48.65±1.29	0.51±0.28	2.40±0.15	0.07±0.00	0.25±0.13
	**L2**	0.78±0.08	0.84±0.03	11.75±1.33	48.40±0.95	0.45±0.12	2.17±0.42	0.07±0.01	0.14±0.06
	**Factor**				**F value**				

**Form**	0.12 *ns*	0.82 *ns*	0.24 *ns*	2.54 *ns*	2.28 *ns*	10.16 *ns*	1.12 *ns*	0.60 *ns*
**St**	0.17 *ns*	1.71 *ns*	0.05 *ns*	0.65 *ns*	0.22 *ns*	1.28 *ns*	0.88 *ns*	0.20 *ns*
**Gr**	0.12 *ns*	8.53***	0.09 *ns*	1.96 *ns*	0.05 *ns*	4.94 *ns*	0.86 *ns*	1.63 *ns*
**Form*St**	0.03 *ns*	0.07 *ns*	0.11 *ns*	0.61 *ns*	0.36 *ns*	0.64 *ns*	0.93 *ns*	0.78 *ns*
**Form*Gr**	0.01 *ns*	0.57 *ns*	0.22 *ns*	0.01 *ns*	3.08 *ns*	0.21 *ns*	0.89 *ns*	0.85 *ns*
**St*Gr**	0.11 *ns*	0.15 *ns*	0.04 *ns*	0.70 *ns*	0.81 *ns*	1.89 *ns*	1.11 *ns*	1.70 *ns*
**Form*St*Gr**	0.39 *ns*	0.26 *ns*	0.48 *ns*	0.76 *ns*	0.72 *ns*	3.32 *ns*	1.12 *ns*	1.66 *ns*

Results as reported like means± standard deviation. a–b indicates significant differences ($p < 0.05$) between the same sample during the shelf-life, A-C indicate significant differences ($p < 0.05$) between treatments, X-Y indicate significant differences (Tukey's test; $p \leq 0.05$) between raw and grilled samples. *$p < 0.05$, **$p < 0.01$, ***$p < 0.001$. Form, formulation; Gr, grilling; St, storage.

		C20:1	C20:2	C22:0	C20:5	C22:5	C22:6
		(% Total Fatty Acid)					
		**0 days**					
**Raw samples**	**C**	0.24±0.00	0.29±0.03	0.14±0.01	0.29±0.09	0.35±0.05	0.07±0.01
	**L1**	0.22±0.02	0.32±0.05^{a}	0.17±0.05^{a}	0.38±0.20^{a}	0.28±0.05	0.07±0.02^{a}
	**L2**	0.24±0.02	0.32±0.10	0.18±0.08	0.49±0.32	0.36±0.05	0.11±0.06
		**6 days**					
	**C**	0.24±0.01	0.34±0.05^{A}	0.15±0.03	0.29±0.04	0.39±0.13	0.09±0.01
	**L1**	0.21±0.14	0.27±0.02^{b,B}	0.12±0.03^{b}	0.24±0.07^{b}	0.42±0.08	0.18±0.06^{a}
	**L2**	0.24±0.02	0.20±0.10^{AB}	0.15±0.04^{Y}	0.27±0.06	0.32±0.03^{X}	0.10±0.11
		**9 days**					
	**C**	0.24±0.02	0.42±0.11	0.15±0.01	0.28±0.10	0.35±0.05^{X}	0.07±0.02
	**L1**	0.19±0.12	0.28±0.02^{b,Y}	0.12±0.04^{Y}	0.22±0.04^{Y}	0.37±0.04^{X}	0.18±0.04^{ab}
	**L2**	0.23±0.01	0.29±0.09	0.11±0.10	0.23±0.30	0.30±0.03^{X}	0.05±0.05
		**0 days**					
**Grilled samples**	**C**	0.22±0.03	0.29±0.04	0.19±0.07^{AB}	0.48±0.26	0.27±0.03^{AB}	0.11±0.04
	**L1**	0.23±0.04	0.26±0.02	0.25±0.02^{A}	0.37±0.29	0.07±0.13^{B}	0.04±0.06
	**L2**	0.24±0.01	0.28±0.05	0.11±0.03^{B}	0.29±0.06	0.30±0.03^{A}	0.05±0.02
		**6 days**					
	**C**	0.22±0.01	0.34±0.04	0.16±0.04	0.32±0.15	0.22±0.02	0.08±0.03
	**L1**	0.25±0.02	0.29±0.16	0.11±0.12	0.30±0.38	0.16±0.14	0.06±0.07
	**L2**	0.25±0.00	0.31±0.02	0.12±0.02^{X}	0.28±0.03	0.16±0.14^{Y}	0.06±0.03
		9 days					

**C**	0.22±0.02	0.37±0.03	0.19±0.03	0.42±0.16	0.26±0.03^{Y}	0.10±0.01
**L1**	0.22±0.02	0.29±0.06^{X}	0.15±0.07^{X}	0.34±0.27^{X}	0.23±0.06^{Y}	0.09±0.05
**L2**	0.23±0.02	0.28±0.04	0.18±0.02	0.30±0.02	0.22±0.01^{Y}	0.05±0.01
**Factor**			**F value**			
**Form**	0.83 *ns*	24.05 *ns*	7.43 *ns*	3.83 *ns*	0.72 *ns*	3.00 *ns*
**St**	0.51 *ns*	2.10 *ns*	7.59 *ns*	3.69 *ns*	0.05 *ns*	0.43 *ns*
**Gr**	0.33 *ns*	0.93 *ns*	9.94**	2.70 *ns*	29.38 *ns*	4.53 *ns*
**Form*St**	0.15 *ns*	2.01 *ns*	2.21 *ns*	0.25 *ns*	1.36 *ns*	2.22 *ns*
**Form*Gr**	1.06 *ns*	0.56 *ns*	1.91 *ns*	1.55 *ns*	1.59 *ns*	8.01 *ns*
**St*Gr**	0.08 *ns*	4.76 *ns*	0.23 *ns*	0.56 *ns*	1.93 *ns*	1.35 *ns*
**Form*St*Gr**	0.21 *ns*	6.33**	2.20 *ns*	2.37 *ns*	0.10 *ns*	0.14 *ns*

Results as reported like means± standard deviation. a–b indicates significant differences ($p < 0.05$) between the same sample during the shelf-life, A-C indicate significant differences ($p < 0.05$) between treatments, X-Y indicate significant differences (Tukey's test; $p \leq 0.05$) between raw and grilled samples. *$p < 0.05$, **$p < 0.01$, ***$p < 0.001$. Form, formulation; Gr, grilling; St, storage.
